# Correction: Structural determinants of macrocyclization in substrate-controlled lanthipeptide biosynthetic pathways

**DOI:** 10.1039/d0sc90208j

**Published:** 2020-09-30

**Authors:** Silvia C. Bobeica, Lingyang Zhu, Jeella Z. Acedo, Weixin Tang, Wilfred A. van der Donk

**Affiliations:** Department of Chemistry and Howard Hughes Medical Institute, University of Illinois at Urbana-Champaign 600 South Mathews Avenue Urbana Illinois 61801 USA vddonk@illinois.edu +1-217-244-8533 +1-217-244-5360; School of Chemical Sciences NMR Laboratory, University of Illinois at Urbana-Champaign 505 South Mathews Avenue Urbana Illinois 61801 USA

## Abstract

Correction for ‘Structural determinants of macrocyclization in substrate-controlled lanthipeptide biosynthetic pathways’ by Silvia C. Bobeica *et al.*, *Chem. Sci.*, 2020, DOI: 10.1039/d0sc01651a.

We recently reported the three-dimensional structures of seven lanthipeptides determined by NMR spectroscopy (*Chem. Sci.*, DOI: 10.1039/d0sc01651a). After publication we became aware that the custom written definition of the non-proteinogenic bis-amino acid methyllanthionine (MeLan) for use by the software package Xplor does not sufficiently define the stereochemistry at C3. As a result, a subset of the seven structures contained a single MeLan with incorrect stereochemistry at C3. We rewrote the definition as shown in the updated ESI, redid all calculations using the original NMR data using the new definition, and manually inspected all 20 lowest energy structures to make sure they all had the correct stereochemistry of MeLan. All of the interactions observed in the original publication were still observed and none of the original conclusions were affected. We replaced the Protein Data Bank (PDB) files for prochlorosins 1.1 and 2.1 and cytolysin L using the same PDB identification numbers as in the original publication (PDB 6VHJ, 6VJQ, and 6VGT, respectively). For prochlorosins 2.10 and 2.11, we deposited the new structures with a new PDB ID (PDB 7JVF and 7JU9, respectively) and rendered the old pdb files obsolete. All BMRB codes remained the same except that BMRB 30789 is now associated with PDB 7JU9. Several of the figures in the original paper are affected in subtle ways and we provide new figures with this correction (Fig. 2–6, 12, and 13).

**Fig. 2:**
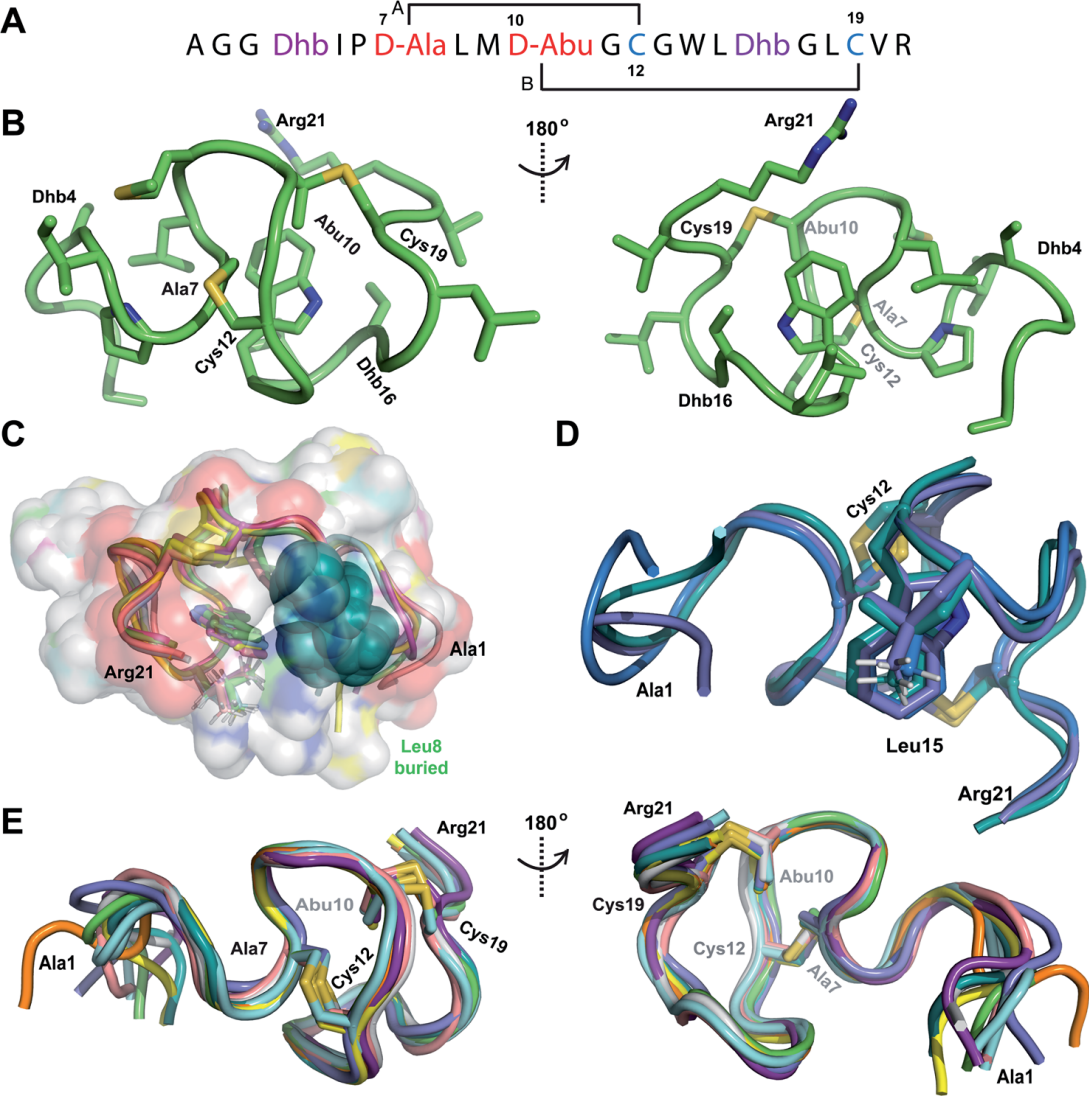
(A) Pcn 2.10 peptide sequence. (B) Representation of the minimum energy structure of the Pcn 2.10 ensemble. Cys and former Ser/Thr amino acids making up the (Me)Lan rings and dehydroamino acids are highlighted. (C) Superimposition of a 5-structure ensemble showing the molecular surface and burial of the hydrophobic side chain of Leu8, colored in green. The N- and C-terminal residues are marked. (D) Superimposition of a 3-structure ensemble showing the alignment of the upfield shifted δ protons of Leu15 over the aromatic side chain of Trp14. Trp14 is almost fully solvent inaccessible. (E) Superimposition of the ensemble of 10 minimum energy structures of prochlorosin 2.10 with the residues involved in thioethers annotated. For a superimposition of the minimum energy 20-structure ensemble of Pcn 2.10, see Fig. S6.

**Fig. 3:**
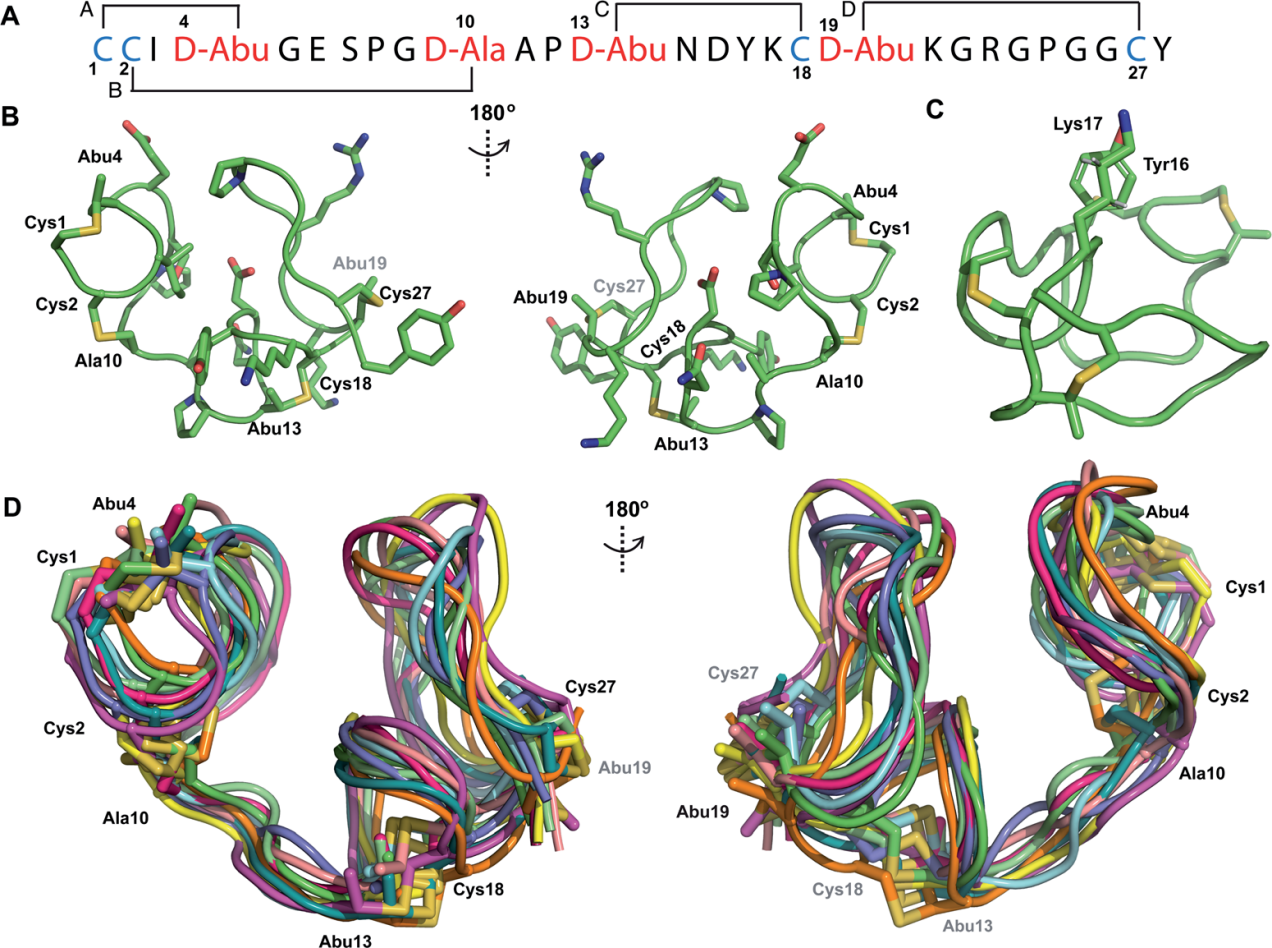
(A) Pcn 2.1 peptide sequence. (B) Representation of the minimum energy structure of the Pcn 2.1 ensemble. (C) Representation of the stacking of the Lys17 γ and δ protons onto the aromatic side chain of Tyr16, which results in an upfield shift of the lysine side chain protons. (D) Superimposition of the ensemble of 10 minimum energy structures for Pcn 2.1. Residues involved in thioether linkages are marked in panels B and D. For a superimposition of the minimum energy 20-structure ensemble, see Fig. S13.

**Fig. 4:**
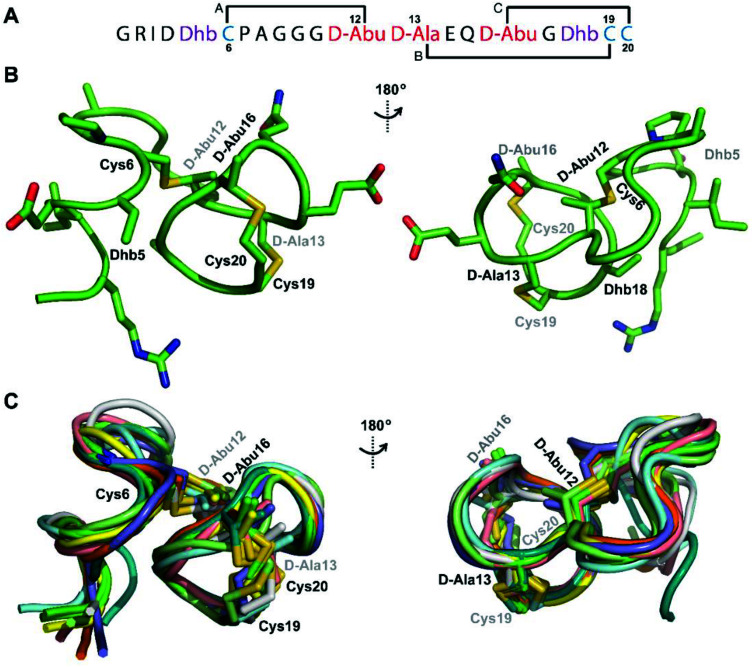
(A) Peptide sequence for Pcn 2.11. (B) Representation of the minimum energy structure of Pcn 2.11. (C) Superimposition of the ensemble of the 10 minimum energy structures for Pcn 2.11. Residues involved in thioether linkages are indicated in both panels. For a superimposition of the minimum energy 20-structure ensemble, see Fig. S18.

**Fig. 5:**
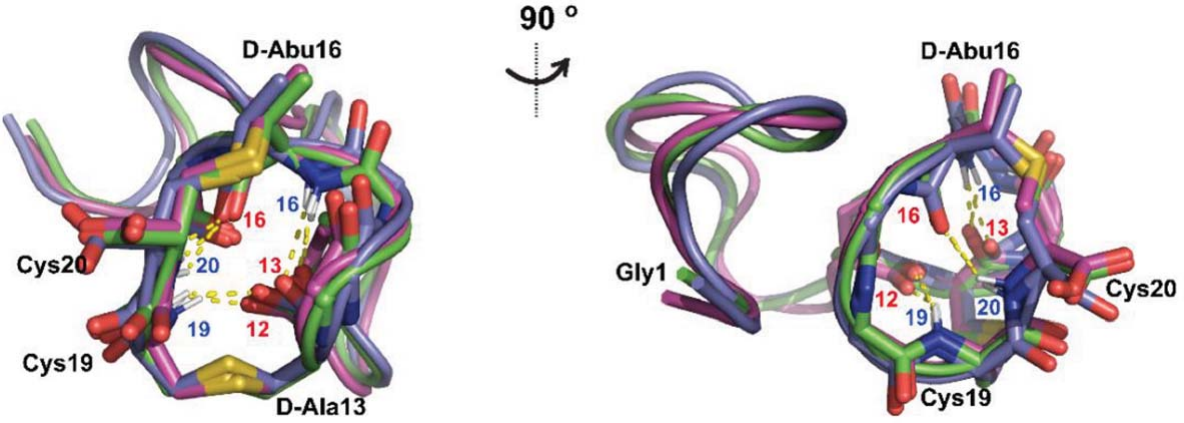
Two views of the hydrogen bonding interactions in Pcn 2.11. The NH donor residue numbers are marked in blue and the oxygen acceptor residue numbers are marked in red. Three structures are shown.

**Fig. 6:**
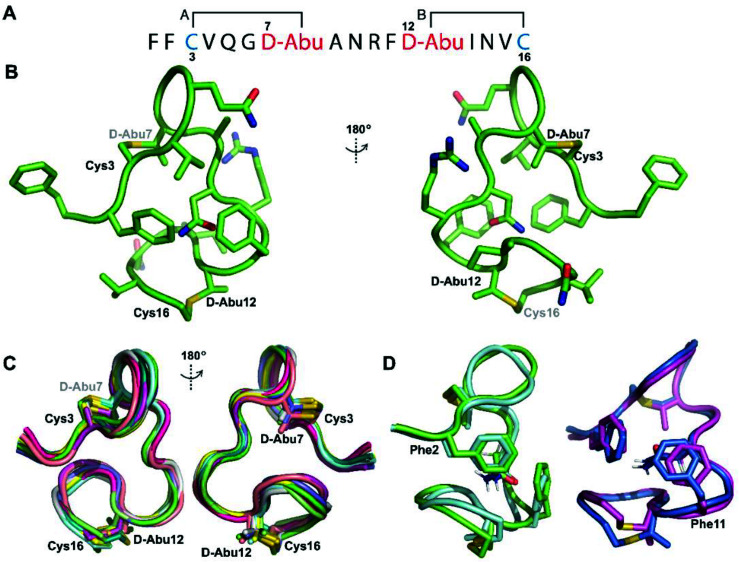
(A) Pcn 1.1 peptide sequence. (B) Representation of the minimum energy structure of prochlorosin 1.1. (C) Superimposition of the ensemble of the 10 minimum energy structures of prochlorosin 1.1. For the ensemble of 20 minimum energy structures, see Fig. S23A. (D) Two views showing the interactions of the β-protons of Asn9 and the aromatic rings of Phe2 and Phe11 illustrated for two structures. For the same panels with the ensemble of the 20 minimum energy structures, see Fig. S23B.

**Fig. 12:**
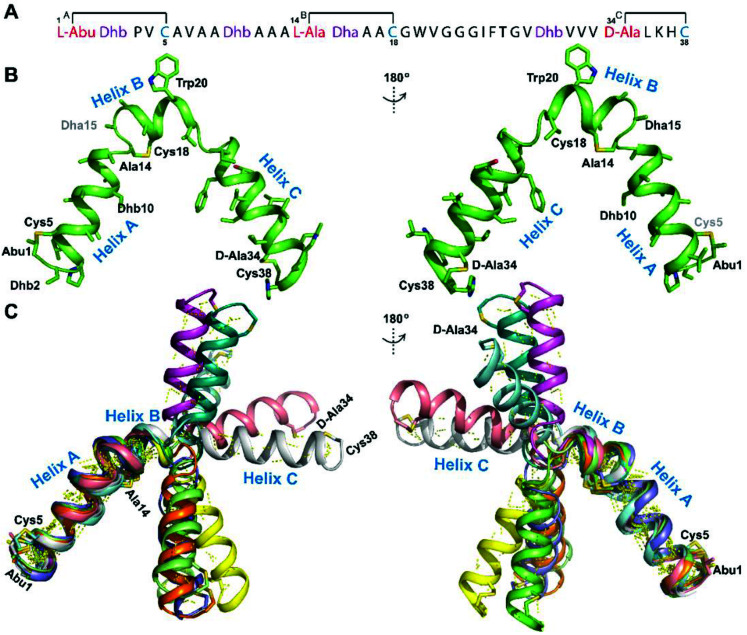
(A) Peptide sequence for CylL_L_′′. (B) Minimum energy structure of CylL_L_′′. Residues involved in thioether linkages and dehydroamino acid residues are marked. The helix at the N-terminus has been termed helix A, the helix spanning the hinge region has been termed helix B, while the C-terminal helix has been named helix C. (C) Superimposition of the minimum energy 10 structure ensemble for CylL_L_′′ with the portion of the peptide corresponding to helix A aligned. Hydrogen bonds are shown in yellow dashed lines.

**Fig. 13:**
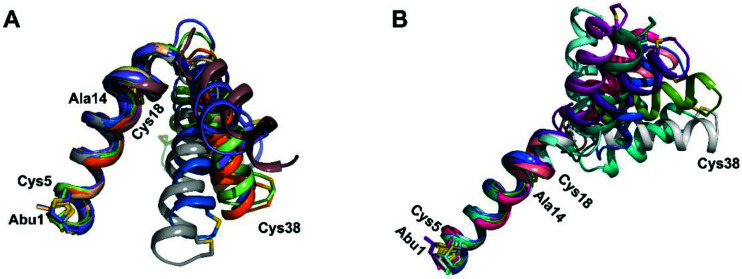
(A) Superimposition of the 10-structure ensemble of CylL_L_′′ that adopts a more compact organization with an acute angle between helices A and C. (B) Superimposition of the 10-structure ensemble of CylL_L_′′ that adopts a more linear conformation with an obtuse angle between helices A and C.

We apologize for this error and thank Prof. Gonzalo Jiménez-Osés (CIC bioGUNE, Spain) for alerting us to the incorrect stereochemistry in a subset of the final structures.

The Royal Society of Chemistry apologises for these errors and any consequent inconvenience to authors and readers.

